# The association between subjective assessment of menstrual bleeding and measures of iron deficiency anemia in premenopausal African-American women: a cross-sectional study

**DOI:** 10.1186/s12905-016-0329-z

**Published:** 2016-08-15

**Authors:** Lia A Bernardi, Marissa S Ghant, Carolina Andrade, Hannah Recht, Erica E Marsh

**Affiliations:** Division of Reproductive Endocrinology and Infertility, Department of Obstetrics and Gynecology, Northwestern University Feinberg School of Medicine, 676 N. St. Clair Street, Suite 1845, Chicago, IL 60611 USA

**Keywords:** Heavy menstrual bleeding, Menorrhagia, African-American, Anemia

## Abstract

**Background:**

Both iron deficiency and iron deficiency anemia are common in the United States with a prevalence amongst women of 12 % and 4 % respectively. These numbers are even higher in African-American women (AAW) and are often a result of heavy menstrual bleeding (HMB). The primary objective of this study was to determine if perceived assessment of menstrual bleeding was associated with objective and subjective measures of anemia in AAW.

**Methods:**

Quantitative cross-sectional pilot study with surveys and venipuncture.

**Results:**

44 premenopausal AAW (mean age 37.9 years ± 9. 4) participated in the study. Iron deficiency was present in 68.2 % of the participants and 18.2 % were anemic. Almost half of the participants reported that their menses were heavy or very heavy, and there was a relationship between perceived heaviness of menstrual flow and anemia (*P* = 0.021). Of the individuals who reported that their menses were heavy or very heavy, 35.0 % were anemic. AAW who reported heavy or very heavy menses had significantly lower hemoglobin (*P* = 0.015), hematocrit (*P* = 0.003), and ferritin (*P* = 0.012) levels, as well as more general (*P* = 0.006) and menses-associated symptoms of anemia (*P* = 0.015) than those who reported normal or light menses.

**Conclusions:**

This pilot study of premenopausal AAW found that a significant percentage of women who report HMB are not only iron deficient, but also anemic. AAW should be educated on the consequences of HMB and counseled to seek care with a women’s health provider when they perceive HMB. More importantly, providers should be aware that when AAW report HMB, evaluation for iron deficiency and anemia are essential.

**Electronic supplementary material:**

The online version of this article (doi:10.1186/s12905-016-0329-z) contains supplementary material, which is available to authorized users.

## Background

Anemia is a global health problem, with over 1.6 billion anemic individuals worldwide [1]. Anemia affects women disproportionately [2]. Globally, approximately 29 % of non-pregnant females aged 15–49 are anemic, which translates to nearly 500 million women [3]. The most common cause of anemia is iron deficiency [4]. Although the prevalence of anemia is higher in underdeveloped countries, there are approximately 5 million individuals affected by iron deficiency anemia in the United States [4]. Up to 12 % of reproductive-aged American women are iron deficient and approximately 4 % suffer from iron deficiency anemia [5]. There is further disparity amongst African-American women (AAW) in whom 19 % have iron deficiency and 12 % have iron deficiency anemia [6]. Heavy menstrual bleeding (HMB), which is a gynecologic problem that affects up to 30 % of premenopausal women, is a common cause of iron deficiency anemia [7, 8].

Despite the fact that healthy premenopausal women with HMB are often able to compensate if anemia develops and thus may avoid complications, women suffering from this condition should undergo medical evaluation as anemia can have a number of negative consequences. Anemia can not only cause impairments in cognitive function and dysfunction in the immune and gastrointestinal systems, but may also significantly impair quality of life and lead to depression, fatigue and impairments in work performance [2, 9, 10]. In addition, anemia is an independent risk factor for morbidity and mortality [2]. While some women do seek outpatient care for abnormal bleeding, making it the cause for up to one-third of gynecologic visits [11], many individuals with HMB do not routinely undergo evaluation [7] and some forgo seeking medical attention until symptoms become severe. These women may ultimately present to the emergency department for initial evaluation [12]. As these individuals may require more urgent and costly evaluation and treatment, receiving care in this manner can have both medical and financial implications. Several factors, including age, race, insurance status, income and geographic location appear to impact whether patients receive gynecologic care in the outpatient setting or the emergency department [12]. However, it is unclear whether specific bleeding patterns influence if and where women seek care.

Women have to recognize that their menstrual bleeding is abnormal before they either see their provider for this indication or respond affirmatively when asked about HMB. Unfortunately, there is not always a strong correlation between an individual’s report of menstrual blood loss and actual amount of measured bleeding [13] or all biomarkers of anemia [14]. In a recent study of women with severe anemia (hemoglobin <5.0 g/dL) due to HMB, 7.8 % reported that their menses were normal. Further, two-thirds of women who had HMB that lasted over six months did not seek medical attention [15]. Although it has been reported that there is not always a strong correlation between subjective vaginal bleeding and objective measures of bleeding [13], it is less clear whether there is a association between perceived HMB and laboratory evidence of anemia. In addition, it has not been fully elucidated if personal assessment of menstrual flow correlates with symptoms of anemia. If an association does exist between perceived assessment of menstrual bleeding and objective and subjective measures of anemia, education to optimize prevention and treatment of anemia in women with HMB becomes even more critical. HMB in AAW is particularly important to investigate, as these individuals have higher rates of HMB [16], and a higher prevalence of iron deficiency and iron deficiency anemia than Caucasian women [5, 6]. Therefore, the primary goal of this pilot study was to determine if perceived assessment of menstrual bleeding was correlated with objective and subjective measures of anemia in AAW. Another aim was to understand whether there was a relationship between reported menstrual patterns and pursuit of medical care.

## Methods

### Participants

The survey used for this cross-sectional pilot study was reviewed and approved by the Institutional Review Board at Northwestern University. Verbal consent was obtained from all study participants. A convenience sample of women was recruited from attendees at an urban community health fair. Women approached a designated area where study personnel offered a brochure describing the study. Following completion of the survey, participants underwent venipuncture for analysis of complete blood counts (CBC) and iron studies. Eligibility criteria included English-speaking women who self-identified as African-American, were over age 18, and reported menstruating. Participants were compensated for their time with gift cards.

### Survey

The survey used in this study was developed by the authors and reviewed by individuals experienced in the fields of gynecology and women’s health. After eligibility was confirmed, participants completed this 44-item anonymous paper survey that asked about demographic information, medical and gynecologic history, symptoms related to anemia and iron deficiency, and healthcare utilization (Additional file 1). Health literacy, defined as “the degree to which individuals have the capacity to obtain, process, and understand basic health information and services needed to make appropriate health decisions” [17], was also assessed as a demographic variable.

Women were specifically asked to classify the heaviness of their menstrual cycle as “very heavy”, “heavy”, “normal”, or “light”. They were also asked to classify their menses by length as <5 days, 5–7 days, or >7 days. A cumulative “general symptoms of anemia” score was calculated based on whether participants reported having to move legs at night to feel comfortable, hair loss, or pica (cravings to eat dirt, clay, starch or ice). A cumulative score of “symptoms of anemia during menses” was also calculated based on how frequently participants reported that they experienced twelve common symptoms of anemia during menses.

### Venipuncture and assays

Following completion of the survey, participants were directed to a station where a trained phlebotomist drew two vials of blood. One vial was drawn in a vacutainer tube containing K_2_EDTA anticoagulant to be used for a CBC. A second vial was drawn in a vacutainer plasma separator tube containing heparin to be used for iron studies. Immediately following the blood draw, samples were spun down using on site centrifuges, and the resultant plasma was stored in a cooler until the end of the fair. The samples were then transported to a large urban academic medical center where the assays were run on the same day as the fair. The Sysmex XE5000 Hematology Analyzer was used to process all CBCs in order to evaluate hemoglobin and hematocrit. Serum iron studies were run using the following kits from Beckman Coulter: direct colorimetric method using TPTZ for iron, the colorimetric method using Nitroso-PSAP for unsaturated iron binding capacity, the immunoturbidimetric method for transferrin and the chemiluminescent sandwich immunoenzymatic method for ferritin. The remaining sera were aliquoted, labeled and stored in a locked freezer in a secured laboratory. Participants were considered anemic if their hemoglobin was less than 11.6 g/dL and iron deficient if serum ferritin was less than 41 ng/mL [18, 19].

### Statistical analyses

Laboratory and survey results were entered into a database and confirmed by one of the study investigators. Frequency analyses were run and distributions were evaluated. Proportions are presented for categorical variables. Means with standard deviations or medians with interquartile ranges (IQR) are presented for continuous variables depending on the normality of the distributions. Chi-square and Mann–Whitney U tests were performed as appropriate to identify associations. A *P*-value of <0.05 was considered to be statistically significant. Statistical analyses were performed with SPSS (PASW version 18) and GraphPad Prism (Version 6.0b).

## Results

### Participant characteristics

There were 44 premenopausal women who completed the survey and agreed to undergo venipuncture. All of the women were African-American with a mean age of 37.9 ± 9.4 years. Overall, 79.5 % of subjects demonstrated adequate health literacy based on a validated single question assessment [20]. Other demographic details are summarized in Table 1.

**Table 1:** Participant Demographics (*n* = 44)

Demographic measures	*n* (%)
Age, y (mean ± SD)	37.9 ± 9.4
Body mass index, kg/m^2^ (mean ± SD)	32.3 ± 8.0
18.5–24.9	8 (18.1)
25–29.9	13 (29.5)
≥30	23 (52.3)
Parity (mean ± SD) (*n* = 43)	1.6 ± 1.5
0	14 (32.6)
1–3	23 (53.5)
4–5	6 (14.0)
Employed	
Yes	21 (47.7)
No	23 (52.3)
Education	
< College	16 (36.3)
≥ College	28 (63.6)
Annual household income	
< $50,000	39 (88.6)
≥ $50,000	5 (11.4)

The majority of these AAW had health insurance, although more than one quarter were uninsured. Just over 50 % of the participants had public insurance. Over 90 % of these AAW reported their health as “very good” “excellent” or “good”, while the remainder stated their health was “fair”. Nearly half of the women described their menses as heavy or very heavy. Over 60 % of participants reported that their menses were shorter than 5 days and approximately one-third stated menses lasted 5–7 days. Only two participants reported that menses lasted more than 7 days, and both of these individuals perceived their menstrual flow as heavy or very heavy. Two participants reported having fibroids and both of these women also classified their menses as heavy or very heavy. Approximately 60 % of these AAW had a gynecologist, and almost 75 % had seen a physician within the last year. Nearly 20 % of participants had a history of seeking outpatient care for heavy vaginal bleeding while only 10 % had visited the emergency room in the past for heavy bleeding.

Table 2 stratifies the participants based on perceived heaviness of menstrual flow and reports the prevalence of various factors related to anemia. Participants who reported heavy or very heavy menses were more likely to have undergone previous evaluation in a clinic or the emergency room for HMB. Both anemia and iron deficiency were more prevalent in participants with heavy or very heavy menses. However, 66.7 % of those who reported normal menses and 33.3 % of those with light menses were also iron deficient.

**Table 2:** Anemia-related Factors Stratified by Reported Menstrual Flow (*n* = 41)

	Light	Normal	Heavy	Very Heavy
	(*n* = 6)	(*n* = 15)	(*n* = 12)	(*n* = 8)
Previous Evaluation for HMB (%)	16.7	0	25.0	50.0
Anemic^a^(%)	0	6.7	41.7	25.0
Iron Deficient^b^ (%)	33.3	66.7	83.3	75.0
General symptoms of anemia score^c^ (mean ± SD)	4.0 ± 0	3.6 ± 0.6	2.5 ± 1.4	3.0 ± 1.2
Symptoms of anemia during menses score^d^ (mean ± SD)	10.8 ± 6.4	8.8 ± 6.0	18.0 ± 9.7	20.8 ± 15.4

^a^Hemoglobin <11.6 g/dL

^b^Ferritin <41 ng/mL

^c^Lower scores translate to more general symptoms of anemia

^d^Higher scores translate to more symptoms of anemia during menses

### Objective and subjective anemia/iron deficiency measures

**Fig. 1:**
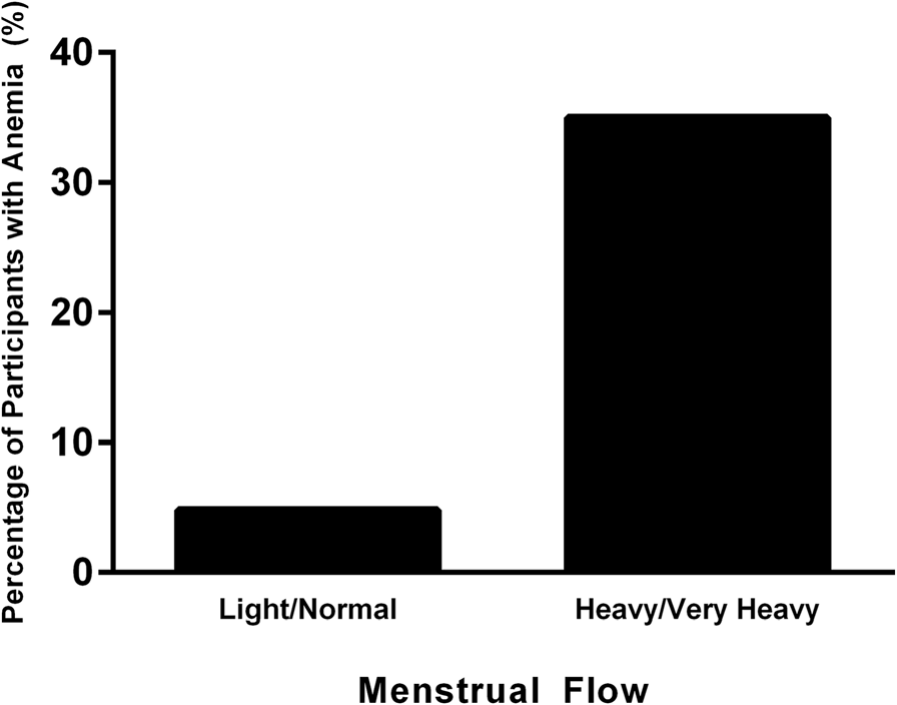
Prevalence of anemia based on perceived heaviness of menstrual flow

Median hemoglobin amongst all participants was 12.9 g/dL (IQR 1.7) and median ferritin was 22.5 ng/mL (IQR 33.2). Overall, 18.2 % of these women were anemic and 68.2 % were iron deficient. Amongst the anemic individuals the median hemoglobin was 10.7 g/dL (IQR 2.3) and the median ferritin was 5.9 ng/mL (IQR 9.6). The women who were anemic were more likely to report heavy or very heavy menses than those who were not anemic (*P* = 0.021). Heavy or very heavy menses were reported in 87.5 % of the anemic women compared with 39.4 % of the non-anemic women. The prevalence of anemia in individuals that reported heavy or very heavy menses was 35.0 % whereas the prevalence of anemia was 4.8 % in those who reported normal or light menses (Fig. 1).

**Fig. 2:**
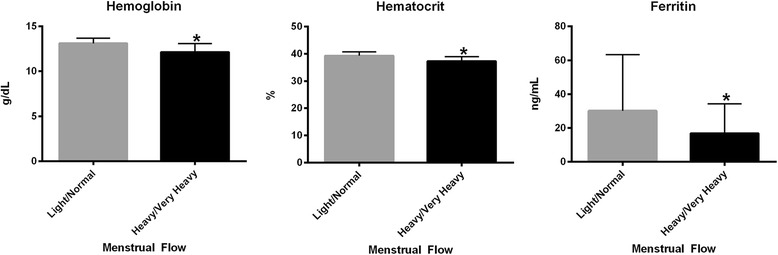
Median (with interquartile ranges) hemoglobin, hematocrit and ferrtin levels by perceived heaviness of menstrual flow

Figure 2 demonstrates median laboratory values by perceived heaviness of menstrual flow. When values were compared between groups, women who reported heavy or very heavy menses had significantly lower hemoglobin (12.2 g/dL versus 13.1 g/dL, *P* = 0.015), hematocrit (37.3 % versus 39.3 %, *P* = 0.003), and ferritin (16.8 ng/mL versus 30.2 ng/mL, *P* = 0.012) levels than those who reported normal or light menses.

Women who described their menses as heavy or very heavy reported more general symptoms of anemia (*P* = 0.006) and more frequent symptoms of anemia during menses (*P* = 0.015) than those who reported normal or light menses.

### Associations

There was an association between reported length of menstrual flow and having a gynecologist (*P* = 0.025). 45.8 % of women who reported that menses lasted less than 5 days had a gynecologist whereas 81.3 % of women who reported that menses lasted 5 days or more had a gynecologist. However, there was not an association between perceived heaviness of menstrual flow and having a gynecologist. Of the AAW who reported heavy or very heavy menses, 40.0 % did not have a gynecologist.

Most of these AAW had not been evaluated for heavy vaginal bleeding in the past, but in women who had been evaluated as outpatients for this complaint, there was no significant association with age, body mass index (BMI), marital status, employment, education, income, insurance status or health literacy. Of the women who had presented to a clinic for heavy vaginal bleeding, half had also been seen in the emergency department (ED) with this complaint (*P* <0.001). There was also no significant association with any of these demographic or socioeconomic factors, insurance status or health literacy in women who had visited the ED for heavy vaginal bleeding.

## Discussion

In this pilot study we found that 68.2 % of urban premenopausal AAW from this convenience sample were iron deficient. Further, 18.2 % of these women were anemic, and there was an association between perceived heaviness of menstrual flow and anemia. Almost half of all participants reported heavy or very heavy menses, and over one-third of these women were anemic. AAW who reported heavy or very heavy menses had significantly lower hemoglobin, hematocrit and ferritin levels, as well as more general and menses-associated symptoms of anemia than those who reported normal or light menses. Although there was an association between length of menses and having a gynecologist, there was not a significant association between perceived heaviness of menstrual flow and having a gynecologist. In addition, there was not an association between seeking care for heavy bleeding as an outpatient or in the ED and any demographic factors, socioeconomic factors, insurance status or health literacy.

The prevalence of anemia in these AAW is markedly higher than the rate of 4 % reported amongst females of all racial and ethnic groups aged 20–49 in the United States [5], and also higher than the reported 12 % prevalence of iron deficiency anemia amongst AAW [6]. Further, almost half of all AAW perceived their menses as heavy or very heavy, which is a higher prevalence than what has been previously reported [8, 16]. These findings in combination with the fact that over one-third of women who reported heavier menses were anemic, whereas less than 5 % of women who reported normal or light menses were anemic, highlight that HMB is a common and potentially serious health problem in premenopausal AAW. Interestingly, women who reported heavy menses had higher rates of anemia and iron deficiency than those who reported very heavy menses. In addition, although the prevalence of iron deficiency was highest in participants who reported heavier menses, approximately two-thirds of women who reported normal menses and one-third of those who reported light menses were also iron deficient. These data suggest that women may be underestimating the heaviness of their menstrual bleeding. Fibroid prevalence was likely underestimated in this study as well. Fibroid incidence amongst AAW is greater than 80 % by age 50 [21]. In this study the prevalence of fibroids was only 4.5 %, suggesting that fibroids were likely under diagnosed in this group despite high rates of HMB.

These data suggest that providers should acknowledge that a patient’s perception of HMB is enough to warrant clinical investigation, but also demonstrate the importance of ensuring that women understand what is considered normal menstrual flow and be educated that heavy menses can lead to iron deficiency anemia. Despite the fact that the correlation between description of menstrual flow and amount of actual bleeding has historically been poor [13], this study confirmed an association between AAW’s perception of HMB and both objective and subjective measures of anemia and iron deficiency. In addition, all but one of the anemic women reported heavy or very heavy menses. Despite these findings, there was no association between perceived menstrual flow and likelihood of having a gynecologist, with over one-third of those who reported heavier menses not having a gynecologist. These study findings suggest that women need to be empowered to feel confident in their ability to determine when they are having HMB and should be encouraged to seek medical care in these situations given that they may be anemic and/or iron deficient. In addition, it is critical that women’s health professionals grasp the magnitude of this problem amongst AAW so that they are prepared to serve as advocates for these individuals.

A main reason that it is important to evaluate women with HMB is to prevent sequelae from iron deficiency and anemia. Treatment of HMB is often possible with appropriate medical evaluation and intervention, as medications as well as surgical procedures can aid in decreasing menstrual flow [22–24]. However, even if treatment of HMB proves challenging, prevention of iron deficiency and anemia is generally possible with fairly conservative measures. Studies have shown that supplemental iron as well as intense dietary programs can aid in improving iron stores [25, 26]. Further, a recent study demonstrated that individuals are interested in learning about the health consequences of iron deficiency and motivated to use a mobile app that aids in improving bioavailable dietary iron [27], suggesting that new technology may make it even easier for individuals to be treated if they suffer from iron deficiency.

In this study, only 19.0 % of women had been evaluated in the clinic for heavy bleeding in the past, despite the high percentage of women who reported HMB. It is important to highlight that AAW with HMB were not more likely to have a gynecologist despite the fact that these women had significantly lower hemoglobin, hematocrit and ferritin levels. Although this is concerning, it may be due to a lack of knowledge regarding etiologies and consequences of HMB. A previous study from our group demonstrated that 63.2 % of AAW could not identify the major causes of HMB when given a list [16]. These findings reinforce the value of educating AAW so that they understand the causes and implications of HMB, as well as the importance of receiving care. As previous data have suggested that demographic factors impact where women seek gynecologic care [12], it was surprising that this study found no correlation between sociodemographic factors and history of outpatient evaluation or ED visits for HMB. Despite a higher prevalence of HMB and anemia in AAW, and the fact that AAW are disproportionally affected by fibroids [28, 29], evidence demonstrates that when compared to Caucasian women, AAW are more likely by two-fold to seek care in an emergency department or hospital outpatient department rather than in a physician’s office [12]. While the reason for this disparity between races is not well understood, it is essential to determine which factors influence choice of setting for gynecologic evaluation in an effort to improve implementation of care.

This prospective pilot study has a number of important strengths. To our knowledge, it is the first to use venipuncture to examine the association between perceived menstrual bleeding and objective measures of anemia in AAW. In addition, this study focuses on a community that is disproportionately affected by HMB and iron deficiency anemia, therefore making this an important group to investigate. In addition, because these individuals were attending a general community fair, they were not specifically seeking out care for their HMB. Rather, these AAW were recruited in their own environment and should represent the general community of urban premenopausal AAW making the results of this study generalizable to a similar patient population.

Although this study highlights a number of important concerns by identifying an association between subjective menstrual assessment and objective measures of anemia in AAW, it is a pilot study and limitations do exist. One limitation is that the survey asked about some but not all causes of anemia. Despite the fact that over 90 % of participants reported being in good health, it is possible that participants were anemic or iron deficient due to diagnoses other than HMB. Therefore, it cannot be confirmed that menstrual bleeding alone contributed to the high rates of anemia and iron deficiency. Another limitation is that a convenience sample was used, which could potentially impact external validity of the study. In addition, this survey was limited to English speaking participants. Further, despite the fact that this is one of the larger studies where venipuncture was performed to assess for anemia in urban premenopausal AAW, the sample size was limited. Finally, as this was a survey study, the results are reliant on subjective responses and self-report of the participants. Although the majority of individuals demonstrated adequate health literacy, recall bias and personal differences in assessment of some of the factors studied exist.

## Conclusions

This pilot study demonstrates that iron deficiency and anemia are common in urban premenopausal AAW and approach levels seen in underserved nations. It also demonstrates that HMB is very common in this cohort. Given that AAW who reported heavier menses were more likely to be anemic, but not more likely to have a gynecologist than those who reported normal or light menses, AAW should be counseled to seek care with a women’s health provider when they perceive HMB. It is particularly important for women who do not routinely see a physician to present for evaluation when they recognize that their menstrual bleeding is heavy. When a woman presents with HMB, clinical investigation is warranted to evaluate for iron deficiency anemia given the demonstrated association between the two. The results from this pilot study can be used to educate and treat women in a way that focuses efforts on health maintenance and prevention. If women with HMB are evaluated prior to developing serious consequences from iron deficiency, more options for treatment are available and frank anemia can be prevented. Ultimately, a larger study examining HMB and anemia in AAW is necessary in order to gain further insight and develop interventions that prevent iron deficiency anemia in this high-risk population of women.

## Abbreviations

AAW, African-American women; CBC, complete blood count; ED, emergency department; HMB: heavy menstrual bleeding

## Additional file

Additional file 1: Study Survey. This is the survey that was completed by individuals who participated in this study. (PDF 108 kb)

## References

[1] McLean E, Cogswell M, Egli I, Wojdyla D, de Benoist B (2009). Worldwide prevalence of anaemia, WHO vitamin and mineral nutrition information system, 1993–2005. Public Health Nutr.

[2] Friedman AJ, Chen Z, Ford P, Johnson CA, Lopez AM, Shander A, Waters JH, van Wyck D (2012). Iron deficiency anemia in women across the life span. J Womens Health.

[3] WHO (2015). The global prevalence of anaemia in 2011.

[4] Miller JL. Iron deficiency anemia: a common and curable disease. Cold Spring Harb Perspect Med 2013;3:pii: a011866. 10.1101/cshperspect.a011866 PMC3685880 23613366

[5] Centers for Disease Control and Prevention (2002). Iron deficiency-- United States, 1999–2000. MMWR Morb Mortal Wkly Rep.

[6] Cusick SE, Mei Z, Freedman DS, Looker AC, Ogden CL, Gunter E, Cogswell ME (2008). Unexplained decline in the prevalence of anemia among US children and women between 1988–1994 and 1999–2002. Am J Clin Nutr.

[7] Fraser IS, Mansour D, Breymann C, Hoffman C, Mezzacasa A, Petraglia F (2015). Prevalence of heavy menstrual bleeding and experiences of affected women in a European patient survey. Int J Gynecol Obstet.

[8] Oehler MK, Rees MC (2003). Menorrhagia: an update. Acta Obstet Gynecol Scand.

[9] Clark SF (2008). Iron deficiency anemia. Nutr Clin Pract.

[10] Côté I, Jacobs P, Cumming DC (2003). Use of health services associated with increased menstrual loss in the United States. Am J Obstet Gynecol.

[11] Abnormal uterine bleeding in peri- and postmenopausal women. When should you see a clinician about excessive or unexpected bleeding? Harv Womens Health Watch 2011;18:4–6. 21305750

[12] Nicholson WK, Ellison SA, Grason H, Powe NR (2001). Patterns of ambulatory care use for gynecologic conditions: A national study. Am J Obstet Gynecol.

[13] Janssen CA, Scholten PC, Heintz AP (1995). A simple visual assessment technique to discriminate between menorrhagia and normal menstrual blood loss. Obstet Gynecol.

[14] Toxqui L, Perez-Granados AM, Blanco-Rojo R, Wright I, Vaquero MP (2014). A simple and feasible questionnaire to estimate menstrual blood loss: relationship with hematological and gynecological parameters in young women. BMC Womens Health.

[15] Nelson AL, Ritchie JJ (2015). Severe anemia from heavy menstrual bleeding requires heightened attention. Am J Obstet Gynecol.

[16] Marsh EE, Brocks ME, Ghant MS, Recht HS, Simon M (2014). Prevalence and knowledge of heavy menstrual bleeding among African American women. Int J Gynaecol Obstet.

[17] Institute of Medicine (US) Committee on Health Literacy; Nielsen-Bohlman L, Panzer AM, Kindig DA, editors. Health Literacy: A Prescription to End Confusion. Washington (DC): National Academies Press (US); 2004. Executive Summary. Available from: http://www.ncbi.nlm.nih.gov/books/NBK216029. Accessed 19 April 2016 25009856

[18] Mast AE, Blinder MA, Gronowski AM, Chumley C, Scott MG (1998). Clinical utility of the soluble transferrin receptor and comparison with serum ferritin in several populations. Clin Chem.

[19] Punnonen K, Irjala K, Rajamaki A (1997). Serum transferrin receptor and its ratio to serum ferritin in the diagnosis of iron deficiency. Blood.

[20] Chew LD, Griffin JM, Partin MR, Noorbaloochi S, Grill JP, Snyder A, Bradley KA, Nugent SM, Baines AD, Vanryn M (2008). Validation of screening questions for limited health literacy in a large VA outpatient population. J Gen Intern Med.

[21] Baird DD, Dunson DB, Hill MC, Cousins D, Schectman JM (2003). High cumulative incidence of uterine leiomyoma in black and white women: ultrasound evidence. Am J Obstet Gynecol.

[22] Lin K, Barnhart K (2007). The clinical rationale for menses-free contraception. J Womens Health.

[23] Chen YJ, Li YT, Huang BS, Yen MS, Sheu BC, Chow SN, Wang PH, Taiwan Association of Gynecology Systematic Review Group (2015). Medical treatment for heavy menstrual bleeding. Taiwan J Obstet Gynecol.

[24] Fergusson RJ, Lethaby A, Shepperd S, Farquhar C (2013). Endometrial resection and ablation versus hysterectomy for heavy menstrual bleeding. Cochrane Database Syst Rev.

[25] Heath AL, Skeaff CM, O'Brien SM, Williams SM, Gibson RS (2001). Can dietary treatment of non-anemic iron deficiency improve iron status?. J Am Coll Nutr.

[26] Patterson AJ, Brown WJ, Roberts DC, Seldon MR (2001). Dietary treatment of iron deficiency in women of childbearing age. Am J Clin Nutr.

[27] Mann D, Riddell L, Lim K, Byrne LK, Nowson C, Rigo M, Szymlek-Gay EA, Booth AO (2015). Mobile phone App aimed at improving iron intake and bioavailability in premenopausal women: a qualitative evaluation. JMIR Mhealth Uhealth.

[28] Stewart EA, Nicholson WK, Bradley L, Borah BJ (2013). The burden of uterine fibroids for African-American women: results of a national survey. J Womens Health.

[29] Richard-Davis G (2013). Uterine fibroid: the burden borne by African American women. J Womens Health.

