# Supplementary material for: The association between subjective assessment of menstrual bleeding and measures of iron deficiency anemia in premenopausal African-American women: a cross-sectional study
Source: BMC Womens Health. 2016 Aug 15;16:50. doi: 10.1186/s12905-016-0329-z (PMC4983800; doi:10.1186/s12905-016-0329-z)
Supplement: Additional file 1: — Study Survey. This is the survey that was completed by individuals who participated in this study. (PDF 108 kb) [file 12905_2016_329_MOESM1_ESM.pdf]

Survey of Symptoms during Your Monthly Period

We are conducting a survey regarding the symptoms you experience during your monthly period. Please help us better understand how your period can affect your quality of life.

Thank you in advance for your participation.

The following set of questions will ask you questions about your medical history. Please check the box you feel is most appropriate. If you do not feel comfortable answering, feel free to skip the next question.

1. I would currently describe my menstrual (monthly periods) status as:

   - ☐ I still have periods
   - ☐ I have undergone menopause and no longer have periods (skip questions 2-6)
   - ☐ I have undergone a hysterectomy and no longer have periods (skip questions 2-6)

2. Are you currently using contraception?

   - ☐ Yes
   - ☐ No

3. If yes, please check the form(s) below:

   - ☐ Birth control pills
   - ☐ Depo Provera injections
   - ☐ IUD – Copper
   - ☐ IUD – Progesterone containing
   - ☐ Nuvaring
   - ☐ Condoms
   - ☐ Other: ______________________

4. Are you currently taking any other medications?

   - ☐ Yes (please list here): ______________________
   - ☐ No

5. On average, how would you describe your periods?

☐ Very heavy
☐ Heavy
☐ Normal
☐ Light

6. On average, how many days do your periods last?

☐ Less than 3
☐ 3-5
☐ 5-7
☐ More than 7

7. If you have undergone menopause or a hysterectomy, what was the year of your last period? ________

8. Have you ever seen a doctor in a clinic for issues related to heavy vaginal bleeding?

☐ Yes
☐ No

9. Have you ever gone to the emergency room for issues related to heavy vaginal bleeding?

☐ Yes
☐ No

10. Overall, how would you rate your health?

☐ Excellent
☐ Very good
☐ Good
☐ Fair
☐ Poor

11. To your knowledge:

	Yes	No	I Don't Know
**a. Do you have any problems with your blood not clotting?**	☐	☐	☐
**b. Do you have any problems with your blood clotting too quickly?**	☐	☐	☐
**c. Do you have uterine fibroids?**	☐	☐	☐
**d. Do you have any problems with your thyroid (this is a gland in the front of your neck)**	☐	☐	☐
**e. Does anyone in your family have sickle cell anemia?**	☐	☐	☐
**f. Have you ever noticed blood in your stools?**	☐	☐	☐
**g. Have you ever noticed black or tarry stools?**	☐	☐	☐

Please identify which of the following conditions you have experienced:

	Yes	No
**12. I have to move my legs a lot at night in order to feel comfortable**	☐	☐
**13. I have experienced hair loss**	☐	☐
**14. I have cravings to eat things like dirt, clay or starch**	☐	☐
**15. I have cravings to eat ice**	☐	☐

The next set of questions asks how you feel during your <u>typical monthly period</u>. Please check the box you feel is most appropriate. There are no right or wrong answers.

	Always	Frequently	Occasionally	Rarely	Never
16. I feel tired during the day	☐	☐	☐	☐	☐
17. I feel dizzy	☐	☐	☐	☐	☐
18. I have low energy	☐	☐	☐	☐	☐
19. I get tired quickly during physical activity	☐	☐	☐	☐	☐
20. I have difficulty remembering things	☐	☐	☐	☐	☐
21. I have difficulty focusing on tasks	☐	☐	☐	☐	☐
22. I catch colds easily	☐	☐	☐	☐	☐
23. I feel irritable	☐	☐	☐	☐	☐
24. I have difficulty completing my work	☐	☐	☐	☐	☐
25. I feel faint or have fainted	☐	☐	☐	☐	☐
26. I experience headaches	☐	☐	☐	☐	☐
27. I have missed work because of how I was feeling	☐	☐	☐	☐	☐

The following set of questions is intended to gain general demographic data. No personal identification will be possible from these questions. Please fill in the blank or check the box where appropriate.

28. Age: _______

29. Height: _______

30. Weight: _______

31. How many times have you been pregnant? _______

32. How many biological children do you have? _______

33. If you have biological children, date when you most recently gave birth: _______

34. Are you employed?

- ☐ Yes
- ☐ No

35. What best describes your health insurance status?

- ☐ No insurance
- ☐ Public (Medicare, Medicaid)
- ☐ Private (i.e. Aetna, Blue Cross, etc.)
- ☐ Military (i.e. VA)
- ☐ Other:

36. Do you have a gynecologist?

- ☐ Yes
- ☐ No

37. When was the last time you were seen by a doctor?

- ☐ Within the last year
- ☐ 2-3 years ago
- ☐ 4-5 years ago
- ☐ 6-10 years ago
- ☐ More than 10 years ago

38. Which of the following best describes your race/ethnicity?

- ☐ African-American/Black
- ☐ Caucasian/White
- ☐ Hispanic/Latino
- ☐ Asian, Hawaiian or Pacific Islander
- ☐ Other: ______________________

39. What is the highest level of education that you have completed?

- ☐ 8th grade or less
- ☐ High school or GED
- ☐ Some college
- ☐ 4-year college degree
- ☐ Master's degree
- ☐ Doctoral degree (MD, JD, PhD)

40. What is your partner status?

- ☐ Single
- ☐ Married
- ☐ Divorced
- ☐ Widowed
- ☐ Cohabitating

41. What is your annual *household* income from all sources?

- ☐ Less than $10,000
- ☐ $10,000 to less than $25,000
- ☐ $25,000 to less than $50,000
- ☐ $50,000 to less than $75,000
- ☐ $75,000 to less than $100,000
- ☐ $100,000 to less than $150,000
- ☐ More than 150,000

42. Do you smoke?

- ☐ Yes
- ☐ No

43. In an average week, how many servings of alcohol do you have? (1 serving = 1 can of beer, 1 glass of wine, or 1 shot of liquor)

- ☐ 0
- ☐ 1
- ☐ 2
- ☐ 3
- ☐ 4
- ☐ >4

44. How confident are you filling out medical forms by yourself?

- ☐ Extremely
- ☐ Quite a bit
- ☐ Somewhat
- ☐ A little bit
- ☐ Not at all
